# Cultural differences in motivational pathways linking coach–athlete relationship and self-reported competitive achievement: a cross-cultural study in east and Southeast Asia

**DOI:** 10.3389/fpsyg.2026.1879038

**Published:** 2026-08-06

**Authors:** Rong Zhang, YongTaek Rhim, Ting Zhang

**Affiliations:** 1 School of Physical Education and Health, Sichuan Technology and Business University, Sichuan, China; 2 Sport and Health Care, Namseoul University, Cheonan-si, Republic of Korea

**Keywords:** basic psychological need satisfaction, coach–athlete relationship, cross-cultural comparison, self-determination theory, self-reported competitive achievement

## Abstract

This study examined whether basic psychological need satisfaction (BPNS) mediated the association between the coach–athlete relationship (CAR) and self-reported competitive achievement and whether these structural relationships differed across two Asian cultural regions. Guided by Self-Determination Theory (SDT), this cross-sectional study included 1,390 university student-athletes from four Asian countries representing East Asia (China and South Korea) and Southeast Asia (Thailand and Malaysia). After establishing full measurement invariance across the two regional groups, multi-group structural equation modeling was conducted while controlling for demographic and sport-related characteristics. Southeast Asian student-athletes demonstrated significantly higher latent levels of CAR and BPNS than their East Asian counterparts. In both groups, CAR was positively associated with BPNS; however, this association was significantly stronger in the East Asian sample (β = 0.53) than in the Southeast Asian sample (β = 0.41). In contrast, the positive association between BPNS and self-reported competitive achievement was significantly stronger in the Southeast Asian sample (β = 0.38) than in the East Asian sample (β = 0.22). Bias-corrected bootstrap analyses further supported significant indirect effects of CAR on self-reported competitive achievement through BPNS in both groups. Overall, the findings suggest that the motivational framework proposed by SDT was applicable across both regional groups, whereas cultural context influenced the relative strength of the motivational pathways linking interpersonal relationships, psychological need satisfaction, and self-reported competitive achievement. By demonstrating systematic within-Asia heterogeneity after establishing measurement equivalence, the present study extends the coach–athlete relationship literature and highlights the importance of considering cultural context when applying relationship-based motivational theories and developing culturally responsive coaching practices.

## 1 Introduction

The quality of the coach–athlete relationship (CAR) is widely recognized as a central interpersonal factor influencing athletes’ psychological development, motivation, and competitive performance. Within Jowett’s (2025) 3Cs framework, CAR is conceptualized as a multidimensional construct comprising commitment, closeness, and complementarity, reflecting the extent to which coaches and athletes maintain enduring commitment, mutual trust, and cooperative interactions. A growing body of sport psychology research has demonstrated that high-quality CAR are associated with greater athlete engagement, psychological well-being, sport persistence, and competitive success. Specifically, supportive coach–athlete dynamics have been linked to reduced stress and burnout risk, higher self-esteem and life satisfaction, stronger sport commitment, and sustained participation across youth, university, and professional sport contexts (Li et al., 2025; Sheridan et al., 2014). Additional evidence further indicates that the quality of the CAR serves as a critical predictor of athletes’ training passion, psychological resilience, and subjective performance satisfaction across diverse competitive settings (Özşaker et al., 2016). Coaches not only provide technical instruction but also shape athletes’ motivational experiences through emotional support, communication quality, and interpersonal interactions. Consequently, understanding how CAR contribute to athletic development has become a major focus in contemporary sport psychology (Jowett et al., 2017; Powers et al., 2020).

Self-Determination Theory (Deci and Ryan, 2000) provides one of the most influential theoretical frameworks for explaining the psychological processes through which interpersonal environments influence behavioral outcomes. SDT proposes that individuals function optimally when three basic psychological needs – autonomy, competence, and relatedness – are satisfied. Within sports settings, coaches play a critical role in facilitating or undermining these psychological needs. Early taxonomies of coaching behavior have also established that interpersonal support and relational quality are core dimensions of effective coaching practice that directly shape athletes’ motivational experiences (Mageau and Vallerand, 2003). Athletes who perceive supportive coaching environments generally report greater need satisfaction, psychological well-being, and performance, whereas controlling coaching behaviors are associated with need frustration (Slemp et al., 2024; Zhu et al., 2024), burnout, and maladaptive outcomes (Gustafsson et al., 2017). Accordingly, numerous empirical studies have supported the proposition that basic psychological need satisfaction (BPNS) constitutes an important psychological mechanism linking CAR with positive athlete outcomes (Choi et al., 2013; Zhang and Rhim, 2024).

Although SDT emphasizes the universality of basic psychological needs, increasing evidence suggests that the ways in which interpersonal relationships satisfy these needs may vary across sociocultural contexts (Du and Ning, 2024). For instance, comparative studies of East Asian sport contexts have documented cross-national variation in how coaching behaviors are perceived and how they map onto athletes’ psychological need satisfaction (Yan et al., 2026). Psychological need satisfaction may represent a universal motivational process, whereas the interpersonal conditions through which these needs are fulfilled are likely to be culturally shaped. Consequently, researchers have increasingly argued that sport motivation should be interpreted within broader cultural and relational contexts rather than assuming identical psychological mechanisms across societies.

This issue is particularly relevant within Asia. Although Asian societies are frequently discussed as representing collectivistic cultures, considerable heterogeneity exists in interpersonal norms, authority structures, and social relationships across different Asian regions. While localized validation studies and single-country investigations have advanced understanding of coach–athlete dynamics in specific Asian contexts (Yang and Jowett, 2010), previous sport psychology research has often treated Asia as a single cultural category, potentially overlooking meaningful regional variation in how athletes perceive coaching relationships and derive psychological support. Such simplification may limit the explanatory power and cross-cultural generalizability of existing motivational models.

Cultural psychology provides a useful framework for understanding these differences, with established theoretical models linking cultural value orientations to patterns of interpersonal interaction, authority dynamics, and relational experience (Tyler et al., 2000). Triandis (1993) proposed that collectivism varies along vertical and horizontal dimensions. Societies characterized by relatively higher levels of vertical collectivism generally emphasize hierarchical relationships, authority, and role obligations, whereas horizontal collectivism places greater emphasis on interpersonal equality, cooperation, and harmonious relationships. Similarly, Hofstede (2001) suggested that societies differ in power distance, reflecting the extent to which unequal authority relationships are accepted within social systems.

These theoretical perspectives may have important implications for CAR. East Asian societies, including China and South Korea, have often been characterized as relatively higher in vertical collectivist orientations than many Southeast Asian contexts. Within such environments, coaching relationships may be more strongly embedded in hierarchical authority structures, and athletes may place greater emphasis on discipline, role expectations, and competence development, with motivational pathways shaped by both psychological need satisfaction and normative obedience to authority (Rathwell et al., 2026). By contrast, many Southeast Asian societies are generally described as placing relatively greater emphasis on interpersonal harmony, emotional support, and cooperative relationships, although considerable variation also exists both within and across countries. Consequently, athletes from different cultural contexts may interpret similar coaching behaviors differently; cross-cultural variation has also been documented in the congruence between coach and athlete perceptions of relational quality and efficacy beliefs (Stephen et al., 2022; Storm and Svendsen, 2023), resulting in heterogeneous associations between CAR and psychological need satisfaction across contexts.

Importantly, these theoretical perspectives should not be interpreted as suggesting that East Asia and Southeast Asia represent culturally homogeneous regions. Considerable diversity exists within each country and across sporting environments. Rather, regional classifications provide a useful contextual framework for examining potential cultural variation in interpersonal processes. Individual athletes may differ substantially in their cultural orientations regardless of national background. Therefore, the present study considers cultural region as a contextual characteristic rather than a direct measure of individual cultural values.

Despite substantial progress in the literature, several important research gaps remain. First, most previous studies examining CAR have been conducted within single-country samples or Western contexts, with much of the existing evidence on relational and motivational dynamics drawn from European, North American, and Australian athlete populations, limiting the generalizability of existing findings to Asian sport settings (Vella et al., 2013; Turnnidge and Côté, 2018). Second, cross-cultural studies have frequently aggregated Asian countries into a single cultural category, despite theoretical and empirical evidence suggesting important regional differences in interpersonal norms and motivational processes. Third, although SDT proposes that psychological need satisfaction represents a central mechanism linking interpersonal relationships and adaptive outcomes, relatively few studies have examined whether the structural relationships among CAR, BPNS, and self-reported competitive achievement differ across Asian cultural contexts using rigorous multi-group structural equation modeling following measurement invariance testing. Furthermore, previous studies have rarely compared East Asian and Southeast Asian university student-athletes within the same analytical framework. Addressing these gaps may contribute to both theory and practice. From a theoretical perspective, examining whether the structural relationships among CAR, BPNS, and self-reported competitive achievement vary across cultural contexts can advance understanding of the boundary conditions of S within sport psychology. From an applied perspective, identifying culturally contingent relationship patterns may assist coaches and sport practitioners in developing coaching strategies that are sensitive to different interpersonal and organizational contexts.

Therefore, the present study investigated the relationships among CAR quality, BPNS, and self-reported competitive achievement among university student-athletes from four Asian countries representing two cultural regions: East Asia (China and South Korea) and Southeast Asia (Thailand and Malaysia). Based on SDT, the CAR framework, and theories of cultural variation, the following hypotheses were proposed:

H1. CAR quality is positively associated with athletes’ BPNS.

H2. BPNS is positively associated with self-reported competitive achievement.

H3. Cultural region moderates the associations between CAR quality, BPNS, and self-reported competitive achievement, such that the strengths of the structural relationships differ between East Asian and Southeast Asian student-athletes.

## 2 Method

### 2.1 Study design and sampling

This cross-sectional study employed a stratified convenience sampling design to investigate the associations among the CAR, BPNS, and competitive achievement among university student-athletes from East Asia and Southeast Asia. Stratification was based on country and cultural region to ensure balanced representation across the four participating countries: China and South Korea (East Asia), and Thailand and Malaysia (Southeast Asia). Participants were recruited from 12 universities (three universities in each participating country) through collaborations with university athletic departments and coaching staff. After ethical approval had been obtained, coaches distributed an online questionnaire to eligible student-athletes during regular training sessions. Participation was voluntary and anonymous, and informed consent was obtained electronically before questionnaire completion. A total of 1,450 questionnaires were distributed, of which 1,426 were returned (response rate = 98.34%). Following data screening, 36 questionnaires were excluded, including 18 with substantial missing data, 12 exhibiting invariant response patterns (e.g., identical responses across all questionnaire items), and 6 that did not meet the predefined eligibility criteria. The final analytical sample comprised 1,390 student-athletes, representing a valid response rate of 97.48%.

### 2.2 Participants

The final sample consisted of 1,390 university student-athletes from four Asian countries, including 354 participants from China, 348 from South Korea, 288 from Thailand, and 400 from Malaysia. Based on cultural region, 702 athletes were classified into the East Asian group and 688 into the Southeast Asian group. The sample included 795 males (57.2%) and 595 females (42.8%), with a mean age of 20.9 ± 1.7 years. Participants represented a broad range of competitive sports, including basketball, football, volleyball, athletics, badminton, table tennis, swimming, martial arts, and other organized sports. Overall, 873 athletes (62.8%) participated in team sports, and 517 (37.2%) participated in individual sports. Most participants were members of university varsity teams and had competitive experience at the university or provincial level, indicating regular engagement in organized competitive sport. Participants were eligible if they (a) were currently enrolled as university student-athletes, (b) had received regular coaching for at least 1 year, (c) had participated in at least one organized competition during the previous 12 months, and (d) provided informed consent. Questionnaires were excluded if they contained substantial missing data, exhibited invalid response patterns, or failed to satisfy the eligibility criteria. Demographic characteristics by country and cultural region are presented in Table 1. Independent-samples analyses indicated no significant between-group differences in gender distribution or age (*p* > 0.05), supporting the demographic comparability of the East Asian and Southeast Asian samples for subsequent cross-cultural analyses.

**TABLE 1:** Demographic and sport characteristics of participants by cultural region.

Region	Country	*N*	Male *n* (%)	Female *n* (%)	Age (M ± SD)	Team sports *n* (%)	Individual sports *n* (%)
East Asia	China	354	195 (55.1)	159 (44.9)	20.4 ± 1.8	219 (61.9)	135 (38.1)
	South Korea	348	216 (62.1)	132 (37.9)	21.1 ± 1.5	197 (56.6)	151 (43.4)
	Subtotal	702	411 (58.5)	291 (41.5)	20.7 ± 1.7	416 (59.3)	286 (40.7)
Southeast Asia	Thailand	288	160 (55.6)	128 (44.4)	20.8 ± 1.6	164 (56.9)	124 (43.1)
	Malaysia	400	224 (56.0)	176 (44.0)	21.3 ± 1.7	293 (73.3)	107 (26.7)
	Subtotal	688	384 (55.8)	304 (44.2)	21.1 ± 1.7	457 (66.4)	231 (33.6)
Total		1390	795 (57.2)	595 (42.8)	20.9 ± 1.7	873 (62.8)	517 (37.2)

### 2.3 Measurement instruments

All measurement instruments were translated and culturally adapted following standardized forward–backward translation procedures to ensure conceptual and semantic equivalence across the four participating countries.

#### 2.3.1 Coach–athlete relationship

The CAR was assessed using the Coach–Athlete Relationship Questionnaire (CART-Q). Consistent with the 3Cs model proposed by Jowett (2007), the questionnaire comprises three dimensions – commitment, closeness (intimacy), and complementarity – and contains 10 items. Responses are rated on a 7-point Likert scale ranging from 1 (strongly disagree) to 7 (strongly agree), with higher scores indicating a higher perceived quality of the CAR.

Confirmatory factor analysis (CFA) supported satisfactory construct validity across both cultural groups. For the East Asian subsample, the model demonstrated acceptable fit: χ^2^/df = 3.249, TLI = 0.941, GFI = 0.953, CFI = 0.968, SRMR = 0.043, RMSEA = 0.051, NFI = 0.957, with standardized factor loadings ranging from 0.625 to 0.863. For the Southeast Asian subsample, model fit was also acceptable: χ^2^/df = 2.88, TLI = 0.933, GFI = 0.929, CFI = 0.952, SRMR = 0.052, RMSEA = 0.061, NFI = 0.957, with standardized factor loadings ranging from 0.595 to 0.780. Internal consistency was good in both groups, with Cronbach’s α coefficients of 0.89 for the East Asian sample and 0.85 for the Southeast Asian sample.

#### 2.3.2 Basic psychological need satisfaction

BPNS was measured using the satisfaction subscale of the Basic Psychological Needs Satisfaction and Frustration Scale (BPNSFS; Bartholomew et al., 2011). The subscale includes 12 items assessing satisfaction with the three core basic psychological needs specified in SDT: autonomy, competence, and relatedness. Responses are scored on a 5-point Likert scale from 1 (strongly disagree) to 5 (strongly agree), with higher scores reflecting greater overall psychological need satisfaction.

CFA results supported robust construct validity in both subsamples. For the East Asian group, model fit was excellent: χ^2^/df = 2.951, GFI = 0.952, CFI = 0.980, SRMR = 0.043, RMSEA = 0.051, NFI = 0.950, with a Cronbach’s α of 0.863. For the Southeast Asian group, adequate model fit was observed: χ^2^/df = 3.121, GFI = 0.952, CFI = 0.960, SRMR = 0.053, RMSEA = 0.063, NFI = 0.950, with a Cronbach’s α of 0.847.

#### 2.3.3 Self-reported competitive achievement

Self-reported competitive achievement was operationalized as participants’ self-reported number of official competitive awards obtained during the preceding 12 months. This measure provides a standardized indicator of recent competitive outcomes while remaining feasible for large-scale cross-cultural survey research.

Because opportunities for obtaining official competition awards may vary across sport disciplines, national competition systems, and institutional contexts, this indicator was interpreted as reflecting one aspect of recent competitive achievement rather than a comprehensive measure of athletic performance. To reduce the potential influence of these contextual differences, sport type and competitive level were included as control variables in all subsequent analyses.

#### 2.3.4 Control variables

Consistent with previous cross-cultural research on coach–athlete dynamics and competitive outcomes, demographic and sport-related characteristics were collected as covariates, including age, gender, years of formal training, weekly training frequency, sport type (team vs. individual), and competitive level (university vs. provincial).

### 2.4 Translation, procedure, and ethical considerations

All questionnaires were administered in the official language of each participating country. Previously validated language versions were adopted whenever available. Where no validated version was available, a standardized forward–backward translation procedure was undertaken. First, two bilingual experts independently translated the original English questionnaires into the target language. Second, an independent bilingual translator who was blinded to the original version performed back-translation. Finally, discrepancies were reviewed by two sport psychology researchers and one professional translator until conceptual and semantic equivalence was achieved. Prior to the formal survey, a pilot study involving 30 university student-athletes from each participating country was conducted to evaluate item clarity, linguistic comprehensibility, and cultural appropriateness. Minor wording modifications were made where necessary, while no substantive changes to the questionnaire structure were required. This study was approved by the Institutional Review Board of Sichuan Technology and Business University (Approval No. SCGS-2513). Participants were informed of the study objectives, the confidentiality of their responses, and their right to withdraw at any time without penalty before providing written informed consent. All data were anonymized and used solely for research purposes.

### 2.5 Data analysis

Data analyses were conducted using SPSS 25.0 and Mplus 8.0. Descriptive statistics, Pearson correlation analyses, and reliability analyses were performed using SPSS. CFA, measurement invariance testing, and multi-group SEM were conducted using Mplus. Potential common method bias was evaluated using the CLF approach. Following the recommendations of Podsakoff et al. (2003), a common latent factor was incorporated into the measurement model, and model fit differences (ΔCFI, ΔRMSEA, and ΔSRMR) together with the variance explained by the common latent factor were examined to determine whether common method bias substantially influenced the results. Measurement invariance was examined sequentially by testing configural, metric, scalar, and residual invariance across the East Asian and Southeast Asian samples (Chen, 2007; Berry et al., 2010). Invariance was considered acceptable when changes in fit indices satisfied the recommended criteria (ΔCFI ≤ 0.010 and ΔRMSEA ≤ 0.015). Subsequently, multi-group SEM was performed to examine the hypothesized relationships among the CAR, BPNS, and self-reported competitive achievement. Age, gender, years of training, weekly training frequency, sport type, and competitive level were included as control variables. Differences in structural path coefficients between cultural groups were evaluated using chi-square difference tests. Indirect effects were estimated using bias-corrected bootstrap procedures with 5,000 resamples and were considered statistically significant when the corresponding 95% confidence intervals did not include zero.

## 3 Result

### 3.1 Measurement invariance

Before conducting cross-cultural comparisons, measurement invariance of the Coach–Athlete Relationship Questionnaire (CART-Q) and the Basic Psychological Needs Satisfaction Scale (BPNSFS) was examined using multi-group confirmatory factor analysis (MG-CFA). Configural, metric, scalar, and residual invariance models were tested sequentially across the East Asian and Southeast Asian samples. As presented in Table 2, the configural model demonstrated an acceptable overall fit to the data (χ^2^/df = 2.32, CFI = 0.94, RMSEA = 0.050, SRMR = 0.040), indicating that the two cultural groups shared a common factor structure. Subsequent tests supported metric invariance (ΔCFI = −0.01, ΔRMSEA = 0.002), scalar invariance (ΔCFI = −0.01, ΔRMSEA = 0.003), and residual invariance (ΔCFI = −0.006, ΔRMSEA = 0.001). All changes in model fit indices satisfied the recommended criteria (ΔCFI ≤ 0.010; ΔRMSEA ≤ 0.015), supporting full measurement invariance across the two cultural groups. The establishment of full measurement invariance indicated that the measurement properties of the CART-Q and BPNSFS were equivalent across East Asian and Southeast Asian student-athletes, thereby permitting meaningful comparisons of latent means and structural relationships.

**TABLE 2:** Measurement invariance of the CART-Q and BPNSFS across cultural groups.

Model	Constraints	χ^2^	df	CFI	RMSEA	SRMR	Δ CFI	Δ RMSEA
Model 1: Configural	None	487.32	210	0.94	0.050	0.040	–	–
Model 2: Metric	Factor Loadings Equal	502.15	228	0.93	0.052	0.045	−0.01	0.002
Model 3: Scalar	Intercepts + Loadings Equal	530.44	246	0.92	0.055	0.050	−0.01	0.003
Model 4: error variance	Residual Variances Equal	548.71	264	0.91	0.056	0.051	−0.006	0.001

To further examine the potential influence of common method bias, a CLF model was estimated. Compared with the baseline measurement model, the inclusion of the common latent factor resulted in only trivial changes in model fit (ΔCFI = 0.003, ΔRMSEA = 0.002, and ΔSRMR = 0.004), all of which were below the recommended thresholds for indicating substantial method effects. Furthermore, the common latent factor accounted for 10.73% of the total variance. Although this indicates the presence of a modest amount of common method variance, the negligible changes in model fit indices suggest that common method bias was unlikely to have materially affected the substantive findings of the study.

### 3.2 Latent mean comparisons

Following the establishment of full measurement invariance, latent mean comparisons were conducted using the East Asian group as the reference group (latent mean fixed at zero). As shown in Table 3, student-athletes from Southeast Asia showed significantly higher latent mean scores for both the CAR (latent mean = 0.73, *p* < 0.001) and BPNS (latent mean = 0.68, *p* < 0.001) than those from East Asia.

**TABLE 3:** Latent mean and sample characteristic comparisons between East Asian and Southeast Asian student-athletes.

Variable	Indicator	East Asia (*n* = 702)	Southeast Asia (*n* = 688)	Test statistic	*p*	Effect size (d/r)
Gender	Male (%)	58.5%	55.8%	χ^2^ = 1.25	0.264	0.09
Training years	M ± SD	4.28 ± 1.86	3.33 ± 1.77	*t* = 10.34	<0.001	0.55
Training frequency	M ± SD	3.83 ± 0.85	3.23 ± 0.98	*t* = 12.15	<0.001	0.66
Number of self-reported competitive awards	M ± SD	3.95 ± 1.75	2.01 ± 1.30	*t* = 23.15	<0.001	1.24
CAR	M ± SD	46.90 ± 12.52	61.07 ± 9.77	*t* = −23.48	<0.001	1.26
BPNS	M ± SD	43.02 ± 5.68	46.26 ± 3.28	*t* = −13.89	<0.001	0.75

Independent-samples analyses further indicated that East Asian athletes reported significantly longer training experience, higher weekly training frequency, and higher self-reported competitive achievement than Southeast Asian athletes (all *p* < 0.001). In contrast, no significant between-group differences were observed in gender distribution (χ^2^ = 1.25, *p* > 0.05) or age (*p* > 0.05), supporting the demographic comparability of the two cultural groups.

### 3.3 Correlations among study variables

Pearson correlation coefficients for the East Asian and Southeast Asian samples are presented in Tables 4 and 5, respectively. Across both cultural groups, all dimensions of the CAR were positively and significantly correlated with BPNS and its three subdimensions (*p* < 0.05). In the East Asian sample, the overall CAR was moderately positively correlated with BPNS (*r* = 0.57, *p* < 0.001). A comparable relationship was observed in the Southeast Asian sample (*r* = 0.59, *p* < 0.001). Among the three dimensions of the CAR, the association between intimacy and complementarity was stronger in the Southeast Asian sample (*r* = 0.80, *p* < 0.001) than in the East Asian sample (*r* = 0.59, *p* < 0.001). Overall, the observed correlation coefficients supported the hypothesized positive associations among the study variables and provided preliminary evidence for the subsequent structural equation modeling analyses.

**TABLE 4:** Correlation matrix for measured variables in East Asia (*N* = 702).

	1	2	3	4	5	6	7	8
1.Commitment	1	1	1	1	1	1	1	1
2.Intimacy	0.69***							
3.Complementarity	0.51***	0.59***						
4.CAR	0.80***	0.83***	0.80***					
5.Autonomy	0.29**	0.24*	0.43***	0.34***				
6.Competence	0.39***	0.33***	0.52***	0.48***	0.60***			
7.Relatedness	0.51***	0.32***	0.24*	0.33***	0.25**	0.34**		
8.BPNS	0.53***	0.41***	0.57***	0.57***	0.77***	0.78***	0.73***	

*P < 0.05, **P < 0.01, ***P < 0.001

**TABLE 5:** Correlation matrix for measured variables in Southeast Asia (*N* = 688).

	1	2	3	4	5	6	7	8
1.Commitment	1	1	1	1	1	1	1	1
2.Intimacy	0.71***							
3.Complementarity	0.76***	0.80***						
4.CAR	0.89***	0.92***	0.89***					
5.Autonomy	0.42**	0.28*	0.35***	0.35***				
6.Competence	0.25*	0.34***	0.31***	0.29***	0.52***			
7.Relatedness	0.68***	0.75***	0.64***	0.72***	0.39**	0.55***		
8.BPNS	0.55***	0.59***	0.50***	0.59***	0.84***	0.81***	0.78***	

*P < 0.05, **P < 0.01, ***P < 0.001.

### 3.4 Multi-group structural equation modeling

A multi-group SEM was estimated to examine the hypothesized structural relationships among the CAR, BPNS, and self-reported competitive achievement while controlling for age, gender, years of training, weekly training frequency, sport type, and competitive level. The model demonstrated satisfactory fit to the data (χ^2^/df = 2.45, CFI = 0.95, TLI = 0.94, RMSEA = 0.04, SRMR = 0.05). As presented in Table 6, the CAR was positively associated with BPNS in both cultural groups. This association was significantly stronger among East Asian athletes (β = 0.53, *p* < 0.001) than among Southeast Asian athletes (β = 0.41, *p* < 0.001), and the between-group difference was statistically significant (Δβ = 0.12, *p* = 0.004). Likewise, the association between BPNS and self-reported competitive achievement was significantly stronger in the Southeast Asian sample (β = 0.38, *p* < 0.001) than in the East Asian sample (β = 0.22, *p* = 0.003), and the between-group difference was statistically significant (Δβ = −0.16, *p* = 0.012). Bias-corrected bootstrap analyses based on 5,000 resamples indicated statistically significant indirect associations between the CAR and self-reported competitive achievement through BPNS in both cultural groups. The standardized indirect effect was 0.12 (95% CI [0.08, 0.16]) for the East Asian sample and 0.16 (95% CI [0.11, 0.21]) for the Southeast Asian sample, as neither confidence interval included zero. The direct association between the CAR and self-reported competitive achievement remained statistically significant in the East Asian sample (β = 0.15, *p* < 0.001) but was not statistically significant in the Southeast Asian sample (β = 0.08, *p* = 0.145). Although the direct effect was not statistically significant in the Southeast Asian sample, the between-group comparison nevertheless indicated that the strength of this structural path differed significantly between the two cultural groups (Δβ = 0.07, *p* = 0.001), indicating that the direct association was significantly stronger in the East Asian group. The structural model accounted 28% of the variance in BPNS and 11% of the variance in self-reported competitive achievement among East Asian athletes, compared with 17% and 19%, respectively, among Southeast Asian athletes.

**TABLE 6:** Multi-group structural equation model results comparing East Asian and Southeast Asian student-athletes.

Structural path	East Asia β	SE	95% CI	*p*	Southeast Asia β	SE	95% CI	*p*	Δβ	Difference test (*p*)
Direct effects										
CAR → BPNS	0.53	0.04	[0.45, 0.61]	<0.001	0.41	0.05	[0.31, 0.51]	<0.001	0.12	0.004
BPNS → self-reported competitive achievement	0.22	0.07	[0.08, 0.36]	0.003	0.38	0.06	[0.26, 0.50]	<0.001	–0.16	0.012
CAR → self-reported competitive achievement	0.15	0.04	[0.09, 0.21]	<0.001	0.08	0.05	[–0.02, 0.18]	0.145	0.07	0.001
Indirect effect (Bootstrap, 5,000 resamples)										
CAR → BPNS → self-reported competitive achievement	0.12	–	[0.08, 0.16]	<0.001	0.16	–	[0.11, 0.21]	<0.001	–	–
Model explained variance (R2)										
BPNS	0.28	–	–	–	0.17	–	–	–	–	–
self-reported competitive achievement	0.11	–	–	–	0.19	–	–	–	–	–

Overall, the findings provided support for the hypothesized positive associations among the CAR, BPNS, and self-reported competitive achievement, while indicating significant cross-cultural differences in the strength of these structural pathways.

## 4 Discussion

### 4.1 Overview of the findings and theoretical interpretation

This study examined whether the associations among the CAR, BPNS, and self-reported competitive achievement differed between university student-athletes from East Asia and Southeast Asia after establishing cross-cultural measurement invariance. Three principal findings emerged. First, the hypothesized positive associations among CAR, BPNS, and self-reported competitive achievement were supported in both cultural groups. Second, Southeast Asian athletes reported higher perceived levels of CAR quality and BPNS than their East Asian counterparts. Third, although the overall pattern of associations was comparable across groups, the strength of the structural pathways differed significantly: the association between CAR and BPNS was stronger among East Asian athletes, whereas the association between BPNS and competitive achievement was stronger among Southeast Asian athletes.

Beyond confirming the proposed hypotheses, these findings provide further insight into the contextual applicability of relationship-based motivational theories in sport. Rather than suggesting that the motivational processes proposed by SDT operate differently across cultures, the present findings indicate that the overall theoretical structure linking interpersonal relationships, psychological need satisfaction, and competitive achievement was supported in both regional groups. At the same time, the observed differences in path strength suggest that cultural context may function as a boundary condition influencing the relative importance of specific motivational pathways, while leaving the underlying theoretical framework intact. This distinction is theoretically important because it shifts the interpretation from questioning the universality of SDT to understanding how motivational processes may be expressed with different degrees of salience across sociocultural contexts (Rottensteiner et al., 2015; Wekesser et al., 2021).

These findings also contribute to the growing body of cross-cultural sport psychology research by emphasizing that equivalent measurement does not necessarily imply equivalent psychological processes. After establishing full measurement invariance, meaningful comparisons of latent means and structural relationships became possible, allowing the present study to demonstrate that comparable theoretical constructs may nonetheless exhibit different relational patterns across cultural settings. Accordingly, the present findings extend previous research by highlighting the importance of examining not only whether motivational theories are applicable across cultures, but also whether the relative strength of their proposed mechanisms varies across different sporting environments (Da Silva et al., 2025).

Importantly, these interpretations should not be understood as evidence that all athletes within East Asia or Southeast Asia share homogeneous motivational characteristics. Rather, the regional comparisons adopted in the present study represent analytical groupings that capture broad sociocultural contexts rather than individual cultural orientations. Because individual-level cultural values, motivational regulation, and autonomy orientation were not directly assessed, should be interpreted as theoretically informed interpretations rather than empirically verified mechanisms.

### 4.2 Interpreting the cross-cultural differences in motivational pathways

The most theoretically meaningful finding was not that the overall motivational model differed across cultural groups, but that the same overall motivational framework was supported in both groups while the relative strength of individual pathways varied. This pattern suggests that cultural context may influence how motivational processes are expressed rather than whether they occur.

The stronger association between CAR and BPNS observed in the East Asian sample may indicate that supportive CAR are more closely intertwined with athletes’ perceptions of psychological need satisfaction within the sporting contexts represented in this study. Previous cross-cultural research has suggested that interpersonal relationships embedded within relatively stronger hierarchical role structures may carry greater motivational significance because coaches often serve not only as technical instructors but also as important sources of guidance, evaluation, and social support. Under such circumstances, athletes may become more likely to interpret relationship quality as an important context through which autonomy, competence, and relatedness needs are experienced. However, this interpretation should be regarded as a theoretically informed explanation rather than a direct empirical conclusion. The present study did not assess individual cultural values, power distance, or motivational regulation. Consequently, it remains equally plausible that organizational climate, coaching philosophy, institutional expectations, or other contextual characteristics contributed to the stronger association observed in the East Asian sample (Oh et al., 2014).

In contrast, the stronger association between BPNS and self-reported competitive achievement observed in the Southeast Asian sample suggests that once athletes perceive their basic psychological needs as being satisfied, these experiences may translate more readily into competitive outcomes. From the perspective of SDT, need satisfaction facilitates autonomous motivation, persistence, adaptive self-regulation, and sustained engagement, all of which have been linked to improved sport performance (Wang and Lian, 2025; Şenel et al., 2025). The present findings are therefore broadly consistent with previous studies reporting positive associations between need satisfaction and adaptive athletic functioning across diverse sporting settings.

Nevertheless, the present data do not permit conclusions regarding the psychological mechanisms responsible for this stronger pathway. Because autonomous motivation, self-regulation, motivational climate, and emotional well-being were not directly measured, the observed differences should not be interpreted as evidence that athletes from Southeast Asia are inherently more autonomously motivated. Instead, the findings suggest that the performance implications of psychological need satisfaction may be more strongly expressed within the sporting environments represented in the Southeast Asian sample.

Taken together, these findings reinforce the view that cultural context should be understood as influencing the expression rather than the existence of motivational processes. Rather than challenging the cross-cultural applicability of SDT or CAR theory, the present study suggests that the relative contribution of interpersonal relationships and psychological need satisfaction to competitive achievement may vary across sporting environments. Future research incorporating longitudinal designs and direct assessments of cultural values, motivational regulation, and coaching climate will be important for clarifying the mechanisms underlying these observed differences.

### 4.3 Cross-cultural differences in the CAR–BPNS-performance associations

A central finding of the present study was that the structural pathways linking the CAR, BPNS, and self-reported competitive achievement differed significantly across the two cultural groups, despite the overall theoretical model being supported in both samples. Specifically, CAR was positively associated with BPNS in both the East Asian and Southeast Asian groups, and BPNS was positively associated with self-reported competitive achievement in both groups. Specifically, the CAR–BPNS association was significantly stronger in the East Asian sample, whereas the BPNS–self-reported competitive achievement association was significantly stronger in the Southeast Asian sample.

More specifically, the association between CAR and BPNS was stronger among athletes in the East Asian sample, whereas the association between BPNS and competitive achievement was stronger among athletes in the Southeast Asian sample. This pattern suggests that the CAR may play a comparatively more central role in shaping athletes’ perceptions of BPNS within the East Asian sporting contexts represented in this study. In contrast, once BPNS are satisfied, these experiences may translate more strongly into competitive achievement within the Southeast Asian sample. Such findings are consistent with previous cross-cultural sport psychology research indicating that interpersonal relationships and motivational processes may operate with different degrees of salience across sporting environments characterized by different social norms, coaching practices, and organizational expectations.

Bootstrap analyses further demonstrated statistically significant indirect associations between CAR and self-reported competitive achievement through BPNS in both cultural groups. However, the direct association between CAR and self-reported competitive achievement remained statistically significant in the East Asian sample but not in the Southeast Asian sample. These findings were consistent with a partial mediation pattern in the East Asian sample and with an indirect-effect-dominant pattern in the Southeast Asian sample. Nevertheless, because the present study employed a cross-sectional design, these mediation patterns should not be interpreted as evidence of distinct causal mechanisms or temporal ordering among the variables (Maxwell and Cole, 2007). Rather, they indicate that the observed associations are consistent with theoretically plausible motivational pathways that warrant further examination using longitudinal or experimental research designs.

Importantly, the present study did not directly assess motivational regulation, autonomy orientation, external regulation, or individual cultural values. Consequently, explanations involving autonomous motivation, authority compliance, or culturally specific motivational mechanisms should be regarded as theoretically informed interpretations rather than empirically verified processes (Lisinskiene and Lochbaum, 2022). Future research incorporating direct measures of these constructs would be valuable for clarifying the psychological mechanisms underlying the observed cross-cultural differences in the CAR–BPNS–self-reported competitive achievement pathways.

It is also important to acknowledge that the measure of competitive achievement was based on athletes’ self-reported official competition awards obtained during the preceding 12 months. Although this measure captures an important aspect of recent competitive success, opportunities to obtain awards inevitably vary across sport disciplines, competition systems, and institutional contexts. To reduce potential contextual confounding, sport type and competitive level were included as covariates in the structural models, and the overall pattern of structural associations remained substantively unchanged after adjustment, suggesting that the findings were relatively robust. Nevertheless, future studies would benefit from incorporating objective competition records, coach-rated performance, or longitudinal performance indicators to provide a more comprehensive assessment of competitive achievement (McGee and DeFreese, 2018).

Taken together, these findings reinforce the view that cultural context may influence the relative strength of motivational pathways linking CAR, psychological need satisfaction, and self-reported competitive achievement, while leaving the overall theoretical framework proposed by SDT intact. Accordingly, the present study contributes to the literature by demonstrating that the same motivational model may be applicable across cultural contexts, but the relative contribution of its constituent pathways may vary across different sporting environments.

### 4.4 Theoretical contributions

The present study makes several theoretical contributions to the sport psychology literature. First, it extends previous research on the CAR by demonstrated that the strength of the associations among CAR, BPNS, and self-reported competitive achievement differs across two Asian cultural regions after establishing measurement invariance. These findings suggest that the application of relationship-based motivational models may benefit from greater consideration of cultural context. Second, this study contributes to the cross-cultural literature on SDT by showing that the theoretical associations proposed by SDT were supported in both cultural groups, although the magnitude of these associations differed. Rather than suggesting that the basic psychological needs operate differently across cultures, the findings indicate that broader social and cultural contexts may influence how interpersonal relationships are associated with psychological need satisfaction and competitive outcomes (Raho and Nam, 2024). Third, the present findings highlight the importance of examining cultural heterogeneity within Asia. Although East Asia and Southeast Asia were treated as two regional groups in this study, substantial diversity undoubtedly exists within each region. Accordingly, the regional classifications adopted here should be interpreted as analytical categories rather than direct indicators of individual cultural orientations (Chen, 2007). Future research should incorporate individual-level measures of collectivism and motivational regulation to better explain the mechanisms underlying cross-cultural differences in CAR. Finally, by integrating CAR theory (Jeong and Choi, 2022) with SDT (Kim, 2021) in a cross-cultural framework, the present study provides empirical support for the contextual applicability of relationship-based motivational models in sport. The findings encourage future research to examine how interpersonal relationships, psychological need satisfaction, and competitive achievement are associated across different cultural settings using longitudinal and multi-method research designs.

## 5 Conclusion and recommendations

### 5.1 Conclusion

The present study investigated the associations among the CAR, BPNS, and self-reported competitive achievement among university student-athletes from East Asia and Southeast Asia. After establishing cross-cultural measurement invariance, the findings supported a common motivational framework across both regional groups while demonstrating significant differences in the strength of the structural associations. Specifically, supportive CAR were positively associated with psychological need satisfaction, which in turn was positively associated with competitive achievement in both groups. However, the relative contribution of these pathways differed across the two sporting contexts.

These findings extend the application of SDT and CAR theory by demonstrating that cultural context may influence the relative salience of motivational pathways without altering the underlying theoretical framework. Rather than indicating fundamentally different motivational systems, the results suggest that the same relationship-based motivational processes may be expressed with different strengths across sporting environments characterized by different social norms, coaching practices, and organizational contexts. This perspective highlights the importance of viewing cultural context as a boundary condition affecting the expression of motivational processes rather than their existence. From an applied perspective, the findings underscore the importance of developing culturally responsive coaching practices. Coaches and sport organizations should recognize that high-quality CAR and the satisfaction of athletes’ basic psychological needs remain fundamental to athlete development across cultural settings, while the relative emphasis placed on interpersonal support and psychological need fulfillment may require adaptation to local sporting environments. Collectively, the present study provides cross-cultural evidence supporting the contextual application of relationship-based motivational models in competitive sport and offers a foundation for future longitudinal, multi-method, and culturally informed research on athlete development.

### 5.2 Limitations

Despite the contributions of the present study, several limitations should be acknowledged. First, the cross-sectional design precludes causal inference and limits conclusions regarding the temporal ordering among the CAR, BPNS, and self-reported competitive achievement. Although the observed associations were consistent with the proposed theoretical framework, the mediation patterns identified in the structural model should be interpreted as statistical associations rather than evidence of causal mechanisms. Second, competitive achievement was assessed using athletes’ self-reported official competition awards obtained during the preceding 12 months. Although this indicator provides a practical and standardized measure of recent competitive achievement for large-scale cross-cultural research, it captures only one aspect of athletic performance and may be influenced by differences in competition opportunities across sport disciplines, competition systems, and institutional contexts. While sport type and competitive level were statistically controlled and the overall pattern of structural associations remained substantively unchanged, future research should incorporate more comprehensive performance indicators, such as objective competition records, coach-rated performance, and longitudinal performance outcomes. Third, the interpretation of the observed cross-cultural differences was informed by SDT and the broader cross-cultural literature rather than by direct measurement of the proposed explanatory mechanisms. Variables such as autonomous motivation, motivational regulation, coaching climate, and individual cultural values were not assessed. Consequently, the psychological mechanisms discussed in this study should be regarded as theoretically informed interpretations rather than empirically verified processes. Finally, although participants were recruited from four countries, they were grouped into two broader regional categories representing East Asia and Southeast Asia. These regional classifications served as analytical groupings for cross-cultural comparison rather than direct indicators of individual cultural orientations. Considerable cultural diversity undoubtedly exists within each region, and caution should therefore be exercised when generalizing the present findings to all athletes within these broader cultural contexts.

### 5.3 Recommendations for future research

Building on the present findings, several directions for future research deserve further attention. First, longitudinal and experimental designs are needed to clarify the developmental and causal relationships among CAR, psychological need satisfaction, and competitive achievement. Such approaches would provide a more rigorous test of the motivational processes proposed by SDT and help establish the temporal sequence underlying the observed associations. Future studies should incorporate a broader range of performance indicators by combining self-reported measures with objective competition records, coach-rated performance, and longitudinal performance trajectories. A multidimensional assessment of competitive achievement would improve measurement validity and strengthen cross-cultural comparisons across different sporting systems.

Future research should directly assess the psychological and cultural mechanisms that may explain the observed cross-cultural differences. Incorporating constructs such as autonomous motivation, motivational regulation, coaching climate, interpersonal trust, and individual cultural values would enable researchers to examine not only whether motivational pathways differ across cultural settings but also why such differences emerge. Finally, future cross-cultural research should move beyond broad regional comparisons by including a more diverse range of countries and sporting contexts. Employing multilevel analytical approaches that simultaneously consider individual-, team-, organizational-, and country-level influences would provide a more comprehensive understanding of how cultural context shapes CAR and athlete development. Such efforts would further advance culturally informed relationship-based motivational models and contribute to a more nuanced understanding of athlete development across diverse sporting environments.

## Data Availability

The original contributions presented in the study are included in the article/supplementary material, further inquiries can be directed to the corresponding author.
